# Elevated Serum Carboxymethyl-Lysine, an Advanced Glycation End Product, Predicts Severe Walking Disability in Older Women: The Women's Health and Aging Study I

**DOI:** 10.1155/2012/586385

**Published:** 2012-08-29

**Authors:** Kai Sun, Richard D. Semba, Linda P. Fried, Debra A. Schaumberg, Luigi Ferrucci, Ravi Varadhan

**Affiliations:** ^1^Department of Ophthalmology, Johns Hopkins University School of Medicine, Baltimore, MD 21287, USA; ^2^Mailman School of Public Health, Columbia University, New York, NY 10032, USA; ^3^Division of Preventive Medicine, Department of Medicine, Brigham and Women's Hospital, Harvard Medical School, Boston, MA 02215, USA; ^4^Longitudinal Studies Section, Clinical Research Branch, National Institute on Aging, Baltimore, MD 21225, USA; ^5^Department of Medicine, Johns Hopkins University School of Medicine, Baltimore, MD 21287, USA

## Abstract

Advanced glycation end products (AGEs) have been implicated in the pathogenesis of sarcopenia. Our aim was to characterize the relationship between serum carboxymethyl-lysine (CML), a major circulating AGE, and incident severe walking disability (inability to walk or walking speed <0.4 m/sec) over 30 months of followup in 394 moderately to severely disabled women, ≥65 years, living in the community in Baltimore, Maryland (the Women's Health and Aging Study I). During followup, 154 (26.4%) women developed severe walking disability, and 23 women died. Women in the highest quartile of serum CML had increased risk of developing of severe walking disability in a multivariate Cox proportional hazards model, adjusting for age and other potential confounders. Women with elevated serum CML are at an increased risk of developing severe walking disability. AGEs are a potentially modifiable risk factor. Further work is needed to establish a causal relationship between AGEs and walking disability.

## 1. Introduction

Mobility difficulties are common among older adults and are associated with poor quality of life [1], increased need for care, and are predictive of death [2–4]. Understanding the processes that lead to disability is important in order to develop strategies to prevent or delay disability in older adults. Lifestyle factors that may influence the pathway to disability include diet. Diet has been incompletely characterized in relation to the development of disability. Recent studies suggest that advanced glycation end products (AGEs), which are active biomolecules formed by the non-enzymatic covalent binding of sugars with proteins and other molecules, may be related to muscle strength and physical performance [5, 6]. The western diet is high in AGEs, which are formed in high concentrations in foods that are prepared at high temperatures. Thus, some foods are considered an important exogenous source of AGEs. AGEs are thought to be absorbed in the process of digestion, circulate in the blood, and can be deposited in different organs and tissues [7].

Sarcopenia, or loss of muscle strength and muscle mass, is an important factor underlying mobility difficulties such as slow walking speed in older adults [8]. Older adults have increased cross-linking of collagen and deposition of AGEs in skeletal muscle [9]. In aging animals, cross-linking of collagen is associated with increased muscle stiffness, reduced muscle function [10, 11], and accumulation of AGEs [12]. AGEs may also play a role in sarcopenia through upregulation of inflammation and endothelial dysfunction in the microcirculation of skeletal muscle through the receptor for AGEs, or RAGE [13]. AGE-induced cross-linking of is collagen elevated in older adults [14] and has been shown to increase the stiffness of human articular cartilage [15].

The relationship between circulating AGEs and the development of disability has not been characterized in older adults. We hypothesized that women with elevated serum carboxymethyl-lysine (CML), an advanced glycation end product, had an increased risk of developing severe walking disability. To address this hypothesis, we examined the relationship between serum CML concentrations and severe walking disability among older women living in the community.

## 2. Subjects and Methods

### 2.1. Study Population

Subjects in this study were women who participated in the Women's Health and Aging Study I (WHAS I), a population-based study designed to evaluate the causes and course of physical disability in older disabled women living in the community. WHAS I participants were recruited from an age-stratified random sample of women aged 65 years and older selected from Medicare enrollees residing in 12 contiguous-zip-code areas in Baltimore [16]. Women were screened to identify self-reported physical disability that was categorized into four domains. The domains of disability were ascertained in a 20- to 30- minute home interview that included questions related to (1) mobility and exercise tolerance, that is, walking for a quarter of a mile, walking up 10 steps without resting, getting in and out of bed or chairs; (2) upper extremity function, that is, raising your arms up over your head, using your fingers to grasp or handle, lifting or carrying something as heavy as ten pounds; (3) higher functioning tasks (a subset of instrumental activities of daily living, not including heavy housework, that is, using the telephone, doing light housework, preparing your own meals, shopping for personal items); (4) basic self-care tasks (a subset of nonmobility dependent activities of daily living, i.e., bathing or showering, dressing, eating, using the toilet). WHAS I enrolled the one-third most disabled women ages 65 and older, those with disability in two or more domains. Of the 1409 women who met study eligibility criteria, 1002 agreed to participate in the study in 1992. There were no major differences in sociodemographic or reported health characteristics between eligible participants and those who declined to participate [16].

At the 12-month follow-up visit (as baseline for the present study), 879 women returned for followup, of which 580 received a blood draw. CML was measured in 531 women who had walking speed measurements available. 119 had severe walking disability, 412 did not have severe walking disability, and of whom 394 who had at least one follow-up visit thereafter were included in the present study. Laboratory measurements of serum CML were done at the 12-month follow-up visit rather than at enrollment because of a greater availability of serum aliquots from this visit.

### 2.2. Data Collection

Standardized questionnaires were administered in the participant's home by trained interviewers. Race was assessed in a questionnaire as black, white, or other, current smoking as yes or no, and education as 0–8, 9–11, 12, or more than 12 years as the highest level of formal education achieved. Two weeks later, a trained registered full-time study nurse practitioner examined each study participant in her home, using a standardized evaluation of physical performance and physical exam. Approximately 75% of women also consented to phlebotomy performed during a separate visit by a trained phlebotomist who followed a standardized protocol. The definitions for the chronic diseases reported in this study were adjudicated by WHAS coinvestigators based on standardized algorithms that combined information from the questionnaire, physical examination, and physician contact [16]. The Mini-Mental State Examination (MMSE) was administered at enrollment. Further details on the methods and sampling design of the WHAS studies are published elsewhere [16].

 The participant was asked to walk over a 4-meter course for each follow-up visit. Participants were instructed to stand with both feet at the starting line and to start walking after a specific verbal command. Timing began when the command was given. In this test, the subject could use a cane, a walker, or other walking aid, but not the aid of another person. The times to complete the first meter and the entire path were recorded. The test was repeated three times, twice at the woman's usual pace, and once at her fastest possible pace. The speed of the faster of the two usual-pace walks was used in the analyses. The length of the walk expressed in meters divided by the time in seconds was used to calculate the average walking speed [16]. Women were categorized as having severe walking disability based upon being unable to walk or having a walking speed < 0.4 m/sec [17]. The 0.4 m/sec cut-off point was approximately at the top of the lowest quartile in the WHAS population at baseline [18] and has been shown to predict functional dependence [19]. Demographic characteristics, self-rated health, and information about appetite and eating were measured in the WHAS questionnaires. Chronic diseases were adjudicated by WHAS coinvestigators based on the questionnaire, physical examination, and physician contact [16]. Vital status was determined through matching with the National Death Index from the 12-month follow-up visit, 1993–96 through the end of 2000. The study protocol was adherent to the Declaration of Helsinki. The Johns Hopkins University Institutional Review Board approved the study protocol, and written informed consent was obtained from all participants.

### 2.3. Laboratory Studies

Nonfasting blood samples were obtained by venipuncture between 9 AM and 2 PM. Blood samples were delivered to Quest Diagnostics Laboratories (formerly Ciba-Corning Laboratories, Baltimore, MD) on the day of blood drawing for complete blood count and creatinine measurements. Serum creatinine was measured using the Jaffe method. Processing, aliquoting, and freezing were carried out at the Core Genetics Laboratory of the Johns Hopkins University School of Medicine following a standardized protocol. Blood samples were stored continuously at −70°C until the time of analyses of serum carboxymethyl-lysine (CML). CML is well characterized, circulating AGE, and one of the dominant AGEs in tissue proteins [20]. CML was measured using a competitive ELISA (AGE-CML ELISA, Microcoat, Penzberg, Germany) [21]. This assay has been validated [22], is specific, and shows no cross-reactivity with other compounds [21]. Measurements were performed in duplicate according to the protocol of the manufacturers, and the results were averaged. The within-assay and between-assay coefficients of variation for serum CML were 3% and 4%, respectively.

### 2.4. Statistical Analysis

Continuous variables were compared using Wilcoxon rank-sum test. Categorical variables were compared using chi-square tests. Body mass index (BMI) was categorized as underweight (<18.5 kg/m^2^), normal range (18.5–24.9 kg/m^2^), overweight (≥25–29.9 kg/m^2^) and obese (≥30 kg/m^2^) [23]. An MMSE score of <24 was defined as cognitive impairment [24]. Renal insufficiency was defined as estimated glomerular filtration rate of <60 mL/min/1.73 m^2^ using the Modification of Diet in Renal Disease equation of Levey and colleagues [25]. Plasma CML was divided into quartiles, and the cut-off between quartiles was 452.6, 558.8, and 689.1 ng/mL. Grouped-time Cox proportional hazards models [26] were used to examine the associations between serum AGEs and the risk of developing severe walking disability because severe walking disability was determined at six-month intervals. The length of followup in longitudinal analyses was 30 months. Women who died, or refused further participation, or were lost to followup after a follow-up visit were censored according to their severe walking disability status at their last visit in the study. 

During the 30 months following the original 12-month follow-up visit (as the baseline for the present study), there were 23 patients who died, 9 of them died after developing severe walking disability whereas 14 of them died with no severe walking disability until the last follow-up visit. Cox proportional hazards models were used to examine the associations between CML and risk of developing severe walking disability. As a highly significant association between slow walking speed and death has been previously described [27], it is a reasonable assumption that some women developed severe walking disability prior to death. For those who died without developing severe walking disability, first, the multiple imputation technique was used to impute the data and then the complete data was analyzed; second, the severe walking disability status known data only was analyzed with weighting by inverse probability weighting (IPW) on the data [28]; finally, two sensitivity analyses were applied to validate those two methods dealing with the incomplete data: (1) treating all missing data because of death as severe walking disability-free; (2) treating the missing data as severe walking disability developed [29]. The statistical program used was SAS 9.1 (SAS Institute, Cary, NC). The level of significance used in this study was *P* < 0.05. 

## 3. Results

There were 394 women who did not have severe walking disability at baseline had serum carboxymethyl-lysine (CML) measurements available, and had at least one followup involved in the present study. One hundred fifty-four (26.4%) of the women developed severe walking disability during followup with an overall rate of severe walking disability of 14.8 per 100 person-years. The characteristics of women who did and did not develop severe walking disability during followup are shown in Table 1. Women who developed severe walking disability were older and more likely to be affected by congestive heart failure, peripheral artery disease, and diabetes. There were no significant differences between women who developed and those who did not develop walking disability by race, education, current smoking, MMSE score, body mass index, hypertension, coronary artery disease, stroke, depression, osteoarthritis, chronic obstructive pulmonary disease, and renal insufficiency. Compared with the group of lower three quartiles of CML, the patients in the group of highest quartile of CML were more likely to develop severe walking disability.

Univariate Cox proportional hazards models were used to examine the relationship between demographic and health characteristics and the development of severe walking disability (Table 2). Highest quartile of CML, older age, congestive heart failure, peripheral artery disease, and diabetes mellitus were associated with an increased risk of developing severe walking disability. Multivariable Cox proportional hazards model that adjusted for age, congestive heart failure, peripheral artery disease, diabetes mellitus, and renal insufficiency are shown in four scenarios (Table 3).

## 4. Discussion

The present study shows that older community-dwelling women with elevated serum CML are at increased risk of developing severe walking disability. These findings are consistent with a previous, cross-sectional study which showed that elevated plasma CML was associated with slow walking speed in older community-dwelling men and women [6]. The present study extends these findings and shows that elevated CML is an independent predictor of the development of severe walking disability.

These observations are consistent with the hypothesis that AGEs play a role in sarcopenia [13]. Increased AGEs may contribute to increased stiffness in muscle tissue and reduced viscoelastic properties of muscle and thus impair muscle function [9]. Collagen is the main structural component of the interstitial space of skeletal muscle and provides elasticity when muscles contract [30]. Aging muscle is characterized by an increase in pyridinium cross-linking of collagen, which is enzymatically derived, and the non-enzymatic accumulation of cross-linking AGEs. In adults without diabetes, the concentration of pentosidine, a major AGE in tissues, was found in skeletal muscle at greater than two times concentration in older compared with younger adults [9]. 

Tendons transfer the forces generated by muscles to bone and have elastic properties that affect the muscle tendon complex. AGEs are deposited in tendons and can affect their structural properties. In a rabbit model, cross-linking of collagen by nonenzymatic glycation increased the structural stiffness of Achilles tendon [31]. In humans, there was a sevenfold difference in concentrations of pentosidine in the patellar tendon in older compared with younger men [14]. Elevated AGEs are also associated with increased bone rigidity [32] which is thought to occur through cross-linking of collagen [33]. 

AGEs may also affect skeletal muscle and tendons through upregulation of inflammation via binding with RAGE [34]. The carboxymethyl-lysine (CML) adduct of AGEs has been identified as a signal-transducing ligand for RAGE, both* in vitro* and *in vivo* [35]. Ligand binding with RAGE triggers the induction of increased reactive oxygen species, activation of NADPH oxidase, and upregulation of inflammation through NF-*κ*B and other signaling pathways [36, 37]. Inflammatory mediators that are upregulated through AGE and the NF-*κ*B pathway include TNF-*α*, IL-6, and C-reactive protein [38]. 

A limitation of this study is that the results cannot necessarily be extrapolated to men or to less disabled women in the community. There are many different AGEs, and in the present study, CML was the only AGE that was measured in serum. However, other studies have shown that circulating CML has a moderate correlation with other AGEs in the blood such as pentosidine and methylglyoxal derivatives [39]. Serum CML concentrations show a moderate correlation with dietary intake of AGEs in older adults [39]. Serum CML concentrations have been reported to vary with dietary intake of AGEs [40], but these findings have not been corroborated by others [41, 42].

## 5. Conclusions

Older women with elevated plasma CML, an advanced glycation end product, had greater risk of developing severe walking disability. Whether modification of dietary intake of AGEs can influence the pathway to disability in older adults is not known.

## Figures and Tables

**Table 1:** Characteristics of women who developed and did not develop severe walking disability in the women's health and aging study I (*N* = 394).

Characteristic	Incident severe walking disability (*n* = 104)	No incident severe walking disability (*n* = 290)	*P*
Age category (years) (%)			
≤69	17.3	26.2	
70–74.9	22.1	27.6	
75–79.9	14.4	22.1	0.0005
80–84.9	14.4	10.0	
85–89.9	23.1	12.1	
≥90	8.7	2.0	

Race (white) (%)	69.2	75.5	0.21
Education < 12 years (%)	62.1	60.3	0.75
Current smoking (%)	15.4	10.0	0.14
Body mass index (kg/m^2^) (%)			
<18.5	3.2	2.8	
18.5–24.9	27.7	23.2	0.83
25.0–29.9	35.1	36.8	
≥30	34.0	37.2	

MMSE score < 24 (%)	15.4	9.7	0.11
Hypertension (%)	57.7	56.2	0.79
Coronary heart disease (%)	24.0	22.1	0.68
Congestive heart failure (%)	14.4	7.2	0.029
Peripheral artery disease (%)	26.0	15.5	0.018
Stroke (%)	1.0	2.8	0.29
Diabetes mellitus (%)	23.1	11.7	0.005
Depression (%)	15.4	11.4	0.29
Chronic obstructive pulmonary disease (%)	29.8	25.9	0.44
Renal insufficiency (%)	58.4	52.5	0.30
Osteoarthritis (%)	57.7	53.5	0.46
CML highest quartile (%)	33.7	22.4	0.02

**Table 2:** Univariate Cox proportional hazard models for demographic and health characteristics and incidence of severe walking disability in the women's health and aging study I (*N* = 394).

Characteristic	Univariate H.R.	95% C.I.	*P*
Age (years)			
≤69	1.00	—	—
70–74.9	1.18	0.64, 2.18	0.60
75–79.9	0.93	0.46, 1.86	0.83
80–84.9	1.96	0.99, 3.89	0.05
85–89.9	2.35	1.28, 4.34	0.006
≥90	5.04	2.25, 11.26	<0.0001

Race (white)	0.76	0.50, 1.16	0.19
Education < 12 years	1.12	0.75, 1.67	0.58
Current smoking	1.59	0.93, 2.70	0.09
Body mass index (kg/m^2^)^1^			
<18.5	0.98	0.30, 3.25	0.98
18.5–24.9	1.00	—	—
25.0–29.9	0.75	0.45, 1.26	0.28
≥30	0.75	0.45, 1.27	0.28

MMSE < 24	1.59	0.93, 2.70	0.09
Hypertension	1.04	0.71, 1.54	0.84
Coronary heart disease	1.09	0.70, 1.71	0.70
Congestive heart failure	1.87	1.08, 3.24	0.03
Peripheral artery disease	1.74	1.12, 2.72	0.01
Stroke	0.46	0.06, 3.31	0.44
Chronic obstructive pulmonary disease	1.21	0.80, 1.85	0.37
Diabetes mellitus	1.79	1.13, 2.85	0.01
Depression	1.21	0.70, 2.09	0.49
Renal insufficiency	1.18	0.79, 1.75	0.42
Osteoarthritis	1.22	0.83, 1.81	0.32
CML highest quartile	1.68	1.11, 2.52	0.01

^1^For body mass index, 18.5–24.9 kg/m^2^ was used as the reference category.

**Table 3:** Multivariate cox proportional hazard models for serum CML and development of severe walking disability, (*N* = 394)^1^.

Scenario	H.R.^2^	95% C.I.	*P*
(1) All women with missing data had multiple simulations to impute severe walking disability status prior to death.	1.63	1.06, 2.49	0.03
(2) Inverse probability weighting method (IPW)	1.56	1.04, 2.36	0.03
(3) All women with missing data treated as censored, that is, no severe walking disability prior to death.	1.46	0.95, 2.23	0.08
(4) All women with missing data treated as developing severe walking disability prior to death.	1.54	1.04, 2.29	0.03

^1^All models adjusted for age, congestive heart failure, peripheral artery disease, diabetes mellitus, and renal insufficiency.

^2^Highest quartile of serum CML versus lower three quartiles.

## References

[1] Groessl EJ, Kaplan RM, Rejeski WJ (2007). Health-related quality of life in older adults at risk for disability. *American Journal of Preventive Medicine*.

[2] Woo J, Ho SC, Yu AL (1999). Walking speed and stride length predicts 36 months dependency, mortality, and institutionalization in chinese aged 70 and older. *Journal of the American Geriatrics Society*.

[3] Rolland Y, Lauwers-Cances V, Cesari M, Vellas B, Pahor M, Grandjean H (2006). Physical performance measures as predictors of mortality in a cohort of community-dwelling older French women. *European Journal of Epidemiology*.

[4] Newman AB, Simonsick EM, Naydeck BL (2006). Association of long-distance corridor walk performance with mortality, cardiovascular disease, mobility limitation, and disability. *Journal of the American Medical Association*.

[5] Dalal M, Ferrucci L, Sun K, Beck J, Fried LP, Semba RD (2009). Elevated serum advanced glycation end products and poor grip strength in older community-dwelling women. *Journals of Gerontology A*.

[6] Semba RD, Bandinelli S, Sun K, Guralnik JM, Ferrucci L (2010). Relationship of an advanced glycation end product, plasma carboxymethyl-lysine, with slow walking speed in older adults: the InCHIANTI study. *European Journal of Applied Physiology*.

[7] Semba RD, Nicklett EJ, Ferrucci L (2010). Does accumulation of advanced glycation end products contribute to the aging phenotype?. *Journals of Gerontology A*.

[8] Lauretani F, Russo CR, Bandinelli S (2003). Age-associated changes in skeletal muscles and their effect on mobility: an operational diagnosis of sarcopenia. *Journal of Applied Physiology*.

[9] Haus JM, Carrithers JA, Trappe SW, Trappe TA (2007). Collagen, cross-linking, and advanced glycation end products in aging human skeletal muscle. *Journal of Applied Physiology*.

[10] Willems MET, Miller GR, Stauber WT (2001). Force deficits after stretches of activated rat muscle-tendon complex with reduced collagen cross-linking. *European Journal of Applied Physiology*.

[11] Ducomps C, Mauriège P, Darche B, Combes S, Lebas F, Doutreloux JP (2003). Effects of jump training on passive mechanical stress and stiffness in rabbit skeletal muscle: role of collagen. *Acta Physiologica Scandinavica*.

[12] Snow LM, Fugere NA, Thompson LV (2007). Advanced glycation end-product accumulation and associated protein modification in type II skeletal muscle with aging. *Journals of Gerontology A*.

[13] Payne GW (2006). Effect of inflammation on the aging microcirculation: impact on skeletal muscle blood flow control. *Microcirculation*.

[14] Couppé C, Hansen P, Kongsgaard M (2009). Mechanical properties and collagen cross-linking of the patellar tendon in old and young men. *Journal of Applied Physiology*.

[15] Verzijl N, deGroot J, Ben Zaken C (2002). Crosslinking by advanced glycation end products increases the stiffness of the collagen network in human articular cartilage: a possible mechanism through which age is a risk factor for osteoarthritis. *Arthritis and Rheumatology*.

[16] Guralnik JM, Fried LP, Simonsick EM, Kasper JD, Lafferty ME (1995). *The Women’s Health and Aging Study: Health and Social Characteristics of Older Women with Disability*.

[17] Ferrucci L, Harris TB, Guralnik JM (1999). Serum IL-6 level and the development of disability in older persons. *Journal of the American Geriatrics Society*.

[18] Rantanen T, Guralnik JM, Ferrucci L (2001). Coimpairments as predictors of severe walking disability in older women. *Journal of the American Geriatrics Society*.

[19] Tinetti ME, Inouye SK, Gill TM, Doucette JT (1995). Shared risk factors for falls, incontinence, and functional dependence: unifying the approach to geriatric syndromes. *Journal of the American Medical Association*.

[20] Reddy S, Bichler J, Wells-Knecht KJ, Thorpe SR, Baynes JW (1995). N*ε*-(carboxymethyl)lysine is a dominant advanced glycation end product (AGE) antigen in tissue proteins. *Biochemistry*.

[21] Boehm BO, Schilling S, Rosinger S (2004). Elevated serum levels of NE-carboxymethyl-lysine, an advanced glycation end product, are associated with proliferative diabetic retinopathy and macular oedema. *Diabetologia*.

[22] Zhang X, Frischmann M, Kientsch-Engel R (2005). Two immunochemical assays to measure advanced glycation end-products in serum from dialysis patients. *Clinical Chemistry and Laboratory Medicine*.

[23] Folstein MF, Folstein SE, McHugh PR (1975). Mini-Mental State’ a practical method for grading the cognitive state of patients for the clinician. *Journal of Psychiatric Research*.

[24] World Health Organization (1995). Physical status: the use and interpretation of anthropometry. *Report of a WHO Expert Committee*.

[25] Levey AS, Bosch JP, Lewis JB, Greene T, Rogers N, Roth D (1999). A more accurate method to estimate glomerular filtration rate from serum creatinine: a new prediction equation. *Annals of Internal Medicine*.

[26] Prentice RL, Gloeckler LA (1978). Regression analysis of grouped survival data with application to breast cancer data. *Biometrics*.

[27] Newman AB, Simonsick EM, Naydeck BL (2006). Association of long-distance corridor walk performance with mortality, cardiovascular disease, mobility limitation, and disability. *Journal of the American Medical Association*.

[28] Carpenter J, Kenward M (2007). A comparison of multiple imputation and inverse probability weighting for analyses with missing data. *Statistical Methods in Medical Research*.

[29] Dufouil C, Brayne C, Clayton D (2004). Analysis of longitudinal studies with death and drop-out: a case study. *Statistics in Medicine*.

[30] Gosselin LE, Adams C, Cotter TA, Mccormick RJ, Thomas DP (1998). Effect of exercise training on passive stiffness in locomotor skeletal muscle: role of extracellular matrix. *Journal of Applied Physiology*.

[31] Reddy GK (2004). Cross-linking in collagen by nonenzymatic glycation increases the matrix stiffness in rabbit Achilles tendon. *Experimental Diabesity Research*.

[32] Vashishth D, Gibson GJ, Khoury JI, Schaffler MB, Kimura J, Fyhrie DP (2001). Influence of nonenzymatic glycation on biomechanical properties of cortical bone. *Bone*.

[33] Hernandez CJ, Tang SY, Baumbach BM (2005). Trabecular microfracture and the influence of pyridinium and non-enzymatic glycation-mediated collagen cross-links. *Bone*.

[34] Basta G (2008). Receptor for advanced glycation endproducts and atherosclerosis: from basic mechanisms to clinical implications. *Atherosclerosis*.

[35] Kislinger T, Fu C, Huber B (1999). N^*ε*^-(carboxymethyl)lysine adducts of proteins are ligands for receptor for advanced glycation end products that activate cell signaling pathways and modulate gene expression. *The Journal of Biological Chemistry*.

[36] Bierhaus A, Illmer T, Kasper M (1997). Advanced glycation end product (AGE)-mediated induction of tissue factor in cultured endothelial cells is dependent on RAGE. *Circulation*.

[37] Li J, Schmidt AM (1997). Characterization and functional analysis of the promoter of RAGE, the receptor for advanced glycation end products. *The Journal of Biological Chemistry*.

[38] Bierhaus A, Schiekofer S, Schwaninger M (2001). Diabetes-associated sustained activation of the transcription factor nuclear factor-*κ*B. *Diabetes*.

[39] Uribarri J, Cai W, Peppa M (2007). Circulating glycotoxins and dietary advanced glycation endproducts: two links to inflammatory response, oxidative stress, and aging. *Journals of Gerontology A*.

[40] Negrean M, Stirban A, Stratmann B (2007). Effects of low- and high-advanced glycation endproduct meals on macro- and microvascular endothelial function and oxidative stress in patients with type 2 diabetes mellitus. *American Journal of Clinical Nutrition*.

[41] Semba RD, Ang A, Talegawkar S (2012). Dietary intake associated with serum versus urinary carboxymethyl-lysine, a major advanced glycation end product, in adults: the Energetics Study. *European Journal of Clinical Nutrition*.

[42] Šebeková K, Krajčovičová-Kudláčková M, Schinzel R, Faist V, Klvanová J, Heidland A (2001). Plasma levels of advanced glycation end products in healthy, long-term vegetarians and subjects on a western mixed diet. *European Journal of Nutrition*.

